# Supplementary material for: The structural basis for RNA slicing by human Argonaute2
Source: bioRxiv. 2024 Aug 20:2024.08.19.608718. Preprint. [Version 1] doi: 10.1101/2024.08.19.608718 (PMC11370433; doi:10.1101/2024.08.19.608718)
Supplement: Supplement 7 — Table S2. Modeling of complex, related to Figures 1, 2, 3, 4. [file media-7.pdf]

Supplementary Table 2: Modeling of HsAGO2 and nucleic acids

Domain/ Chain ID	Domain/ region	Residue range	Initial Model	PDB template for initial model/chain	Map used for modeling	Modeling algorithm	Changes to initial model	Confidence of modeling	Relative map resolution
**N-term/A**	N-term	23-52	Crystal Structure	4OLA	2	Rigid body fitting	Manual correction, phenix.real_space_refine	Atomic model	3-8 Å
**N/A**	N	53-140	Crystal Structure	4OLA	2	Rigid body fitting	Manual correction, phenix.real_space_refine	Atomic model	6-8 Å
**L1/A**	L1	141-229	Crystal Structure	4OLA	2	Rigid body fitting	Manual correction, phenix.real_space_refine	Atomic model	6-8 Å
**PAZ/A**	PAZ	230-348	Crystal Structure	4OLA	2	Rigid body fitting	Manual correction, phenix.real_space_refine	Atomic model	6-8 Å
**L2/A**	L2	349-444	Crystal Structure	4OLA	2	Rigid body fitting	Manual correction, phenix.real_space_refine	Atomic model	3-8 Å
**MID/A**	MID	445-577	Crystal Structure	4OLA	2	Rigid body fitting	Manual correction, phenix.real_space_refine	Atomic model	3-8 Å
**PIWI/A**	PIWI	578-821, 845-859	Crystal Structure	4OLA	2	Rigid body fitting	Manual correction, phenix.real_space_refine	Atomic model	3-8 Å
**PIWI/A**	PIWI/Eukaryotic Insertion Loop	822-844	Alphafold2	N/A	2	Alphafold2	Manual correction, phenix.real_space_refine	Alphafold2	6-8 Å
**Guide RNA/G**	Guide RNA	1-8	Crystal Structure	6N4O	2	Rigid body fitting	Manual correction, phenix.real_space_refine	Atomic model	3-8 Å
**Guide RNA/G**	Guide RNA	9-21	ChimeraX generated	N/A	2	Rigid body fitting	Manual correction, phenix.real_space_refine	Pseudo-atomic model	3-8 Å
**Target RNA/T**	Target RNA	15-22	Crystal Structure	6N4O	2	Rigid body fitting	Manual correction, phenix.real_space_refine	Atomic model	3-8 Å
**Target RNA/T**	Target RNA	1-14	ChimeraX generated	N/A	2	Rigid body fitting	Manual correction, phenix.real_space_refine	Pseudo-atomic model	3-8 Å
